# Diastolic versus systolic ankle-brachial pressure index using ultrasound imaging & automated oscillometric measurement in diabetic patients with calcified and non-calcified lower limb arteries

**DOI:** 10.1186/s12872-016-0377-1

**Published:** 2016-10-26

**Authors:** Akram M. Asbeutah, Abdullah A. AlMajran, Sami K. Asfar

**Affiliations:** 1 Department of Radiologic Sciences, Faculty of Allied Health Sciences, Kuwait University, P.O.Box 31470, Kuwait, 90805 Kuwait; 2 Department of Community Medicine & Behavioural Sciences, Health Sciences Centre, Faculty of Medicine, Kuwait University, Kuwait, Kuwait; 3 Department of Surgery, Faculty of Medicine, Kuwait University & Vascular Surgery Unit, Mubarak Al-Kabeer Hospital, Ministry of Health, Kuwait, Kuwait

**Keywords:** Ankle brachial pressure index, Peripheral arterial disease, Atherosclerosis, Diastolic ankle brachial pressure index, Systolic ankle brachial pressure index, Diabetic foot

## Abstract

**Background:**

Ankle-brachial pressure index-systolic (ABI-s) can be falsely elevated in the presence of calcified lower limb arteries in some diabetic patients and therefore loses its value in this cohort of patients. We aim at investigating the feasibility of using the diastolic (ABI-d) instead of ABI-s to calculate the ABI in diabetic patients with calcified limb arteries.

**Methods:**

A total of 51 patients were chosen from the diabetic foot clinic. Twenty six of these patients had calcified leg arteries by Duplex scan (Group A) and 25 patients did not have calcifications in their leg arteries (Group B). Twenty five healthy volunteers were enrolled in the study for group C and they were matched with other participants from group B and A in age and sex. ABI measurement was performed using “boso ABI-system 100 machine”. Systolic ABI (ABI-s) and diastolic ABI (ABI-d) were calculated based on bilateral brachial and ankle oscillometric pressures. ABI is considered normal when it is ≥0.9. Repeated measures ANOVA test was used to test for comparing mean scores for ABI-s and ABI-d across the three groups. Statistical significance is considered when *P* < .05.

**Results:**

The mean age of all participants (±SD) was 64.30 ± 7.1 years (range, 50–82 years). ABI-s mean ± SD was 1.3 ± 0.10 (range, 1.18–1.58) in group A patients, 1.07 ± 0.05 (range, 1–1.16) in group B patients, and 1.06 ± 0.05 (range, 1–1.16) in group C volunteers. While ABI-d mean ± SD was 1.07 ± 0.05 (range, 1.1–1.17) in group A patients, 1.06 ± 0.05 (1–1.14) in group B patients, and 1.05 ± 0.04 (range, 1.01–1.14) in group C volunteers. In group A, repeated measures ANOVA test showed statistical significant difference between ABI-s and ABI-d (*P* < 0.001) whereas in group B & C was not (*P* > 0.05).

**Conclusions:**

ABI-d may be helpful and can be used as a complementary measure instead of ABI-s in falsely elevated ABI caused by partial incompressible vessel.

## Background

The prevalence of peripheral arterial disease (PAD) in Europe and North America is approximately 27 million people [1]. In the Gulf region and Middle East, it was shown that patients with acute coronary syndrome (ACS) constitute a high risk group for PAD [2–4]. Unfortunately, despite its prevalence and the associated cardiovascular risks, only 25 % of PAD patients receive active treatment [5]. Many studies showed that by simply measuring the ABI, many patients with unrecognized PAD can be diagnosed. This early detection of PAD by this test is crucial as it has been found that the presence of PAD is an independent predictor of future cardiovascular disease [6, 7].

Since the very beginning of modern noninvasive vascular laboratory studies, the ankle-brachial systolic pressure index (ABI-s) has been a fundamental tool used to detect, screen, and follow up of patients suffering from PAD [8]. Measurement of the ABI can easily be performed in the clinician’s office using a blood pressure cuff and hand-held Doppler device with a vascular probe. Systolic blood pressure is determined in both arms (brachial pressure) and both ankles (anterior and posterior tibial arteries) [9]. The ABI is calculated for each lower limb by dividing the higher of the two tibial systolic pressures by the systolic arm pressure of the same side to get the right and left leg ABIs.

The ABI is very specific and sensitive technique with 95 % accuracy in detecting PAD and an abnormal ABI is considered as a marker of future cardiovascular disease even in the absence of symptoms. [10] ABI value of >0.9 is considered normal and a value of <0.9 suggests significant disease in one or more arteries of the leg. The majority of patients with claudication have ABIs of less than 0.9. Rest pain, critical limb ischemia (CLI), or severe occlusive disease typically occurs with an ABI <0.5, and an ABI of <0.2 is associated with gangrene. However, a normal ABI value does not absolutely rule out the possibility of PAD. Some patients with symptoms suggestive of PAD but have normal or near-normal ABI results. In these patients, an exercise ABI should be conducted [11].

Despite its high accuracy, several technical questions have been raised about using the systolic blood pressure in calculating the index (ABI-s) in patients with calcified and incompressible arteries. Diabetic patients who have end-stage renal disease and those senior diabetic patients are able to give normal or falsely elevated ABI-s (value: 1.3-2). Therefore, using their data nullifies the value of the test for diagnosis and follow up [12].

Several studies demonstrated that ABI measurements with pocket Doppler and automated oscillometric vascular laboratory equipment yield comparable results and can replace each other [9, 13]. The automated oscillometric technique for pressure measurement takes less than 5 min to perform, it has opened a window of opportunity to re-evaluate the traditional ankle pressure measurements in the presence of total or partial arterial incompressibility [14, 15]. With this technique diastolic pressures may be measured appropriately even if the systolic pressure is overestimated or immeasurable [16].

To our knowledge, there is no article in literature about measuring the diastolic pressure to calculate the ABI in patients with diabetes with calcified leg arteries instead of measuring the systolic pressure in the Middle East. We used the automated oscillometric technique to measure systolic and diastolic ABI in diabetic patients who had calcification and those who did not have calcification in their lower limb arteries. In this study, we aimed at evaluating the feasibility of using the diastolic (ABI-d) instead of the systolic (ABI-s) in diabetic patients with calcified incompressible leg arteries. The question was: could ABI-d be used to replace falsely elevated ABI-s in diabetic patients?

## Methods

### Subjects

Patients under the care of the senior consultant vascular surgeon (SA) and attending the diabetic foot clinic who did not complain from any peripheral arterial symptoms (claudication, rest pain or critical limb ischemia) and having palpable pedal pulses, were approached to be part of this study. Fifty one patients were participated and signed an informed consent prior to the study. All subjects were asked to fill a questionnaire including their personnel details, risk factors for PAD, and medication history. All subjects underwent a clinical review and physical examination of both legs by one senior consultant vascular surgeon (SA) upon recruitment before undergoing duplex ultrasound study and ABI measurements. They were first screened for the presence of arterial wall calcification in the lower limb arteries (superficial femoral artery-SFA, popliteal-POP.A & tibial arteries -TAs) by Duplex ultrasound. Twenty six of them were found to have diffuse circumferentially non-compressible calcified leg arteries throughout the SFA, POP. A, and TAs but with no significant hemodynamic stenosis (<30 % diameter lumen reduction) or flow-limiting changes (group A), the other 25 patients were found to have no arterial wall calcifications in their lower limbs arteries (group B). A 25 healthy non-diabetic volunteers matching the age and sex of other groups were enrolled in the study for comparison (group C). The study was approved by the Human Research and Ethics Committee, Health Sciences Centre, Kuwait University.

### Ultrasound imaging

Examinations for the presence of calcified arterial walls were performed with the patient in the supine position. B-mode, colour Doppler ultrasound (CDU) and pulsed Doppler ultrasound (PDU) were performed in both lower limbs using a GE (Voluson E8 Model, USA- Bothel) scanner with a linear 9 MHz transducer. All examinations were performed in a temperature controlled room at 22° ± 2C and before commencing the study, all patients were asked to remove their clothing including undergarments and change into loose clothing gowns to avoid pressure on arteries. Prior to the examination each patient was rested (sitting or lying) for at least 10 min to exclude exercise associated hyperaemia.

A longitudinal plane of the lower limb arteries including SFA, POP.A and TAs was depicted while avoiding pressure to rule out iatrogenic influences on the artery diameter. Investigation proceeded in defined steps: **(1)** B-mode image search for diffuse circumferentially non-compressible calcified leg arteries; **(2)** color Doppler was switched on and an optimal image of lower limb arteries were obtained to demonstrate blood flow with no significant flow-limiting changes; and **(3)** pulsed Doppler sample volume was placed in the lower limb arteries and the peak systolic and end diastolic flow were obtained from the spectral analysis to exclude lower limb arteries stenosis/occlusion. The Doppler angle was set at 60° in lower limb arteries. This is followed by ABI measurement. The examinations were performed by a fully qualified and experienced vascular technologist (AMA).

### Ankle-brachial pressure index (ABI) measurement

Following the duplex ultrasound, the patients were divided into group A with diffuse circumferentially non-compressible calcified lower limb arteries but with no significant or flow-limiting changes, group B with non-calcified lower limb arterial wall, and group C non-diabetic and with non-calcified leg arteries. The patients waited in a sitting position 15 min after the duplex ultrasound procedure. Then they laid on the bed and stayed in a horizontal position for an additional few minutes while receiving explanations about the procedure.

An oscillometric device (boso ABI-system 100; BOSCH & SOHN, Germany) for ABI measurements was used. Before starting with the chosen cohort we tested the machine for reliability and for validation of obtained numbers. For this purpose we randomly chose 8 of them (16 limbs). They were examined by the oscillometric device to calculate the ABI-s and ABI-d. For each subject, the test was repeated three times on the same day, with 5 min intervals.

ABI was measured in a very short time with the push of a button by using four simultaneously applied blood pressure cuffs. Very few measurements were retaken because of patient’s movement or poor positioning. The ABI-s and ABI-d measurements for either leg were calculated by the computer on the basis of the greatest systolic or diastolic arm pressure. ABI (systolic or diastolic) was considered normal when it is ≥0.9 whereas ≥1.2 is considered falsely elevated [10].

### Statistical analysis

Patient data were entered in a Microsoft Excel file version 2013. The data were analyzed using IBM Statistical Package for Social Science (SSPS) for windows version 23.0 Armonk NY. The patient characteristics were analyzed. Interclass Correlation Coefficient (ICC) for the repeated measurements to test for machine reliability and measurements validation were used and interpreted based on their proximity to a value of 1.00. Also 95 % confidence interval (CI) was reported for the repeated measurements. Generalized linear model (GLM) univariate was done to find out the delta (ABI-s minus ABI-d) for each leg across the three groups. Also repeated measures ANOVA test was used to test for comparing mean scores for ABI-s and ABI-d across the three groups (A, B, and C) for each leg separately. Statistical significance is considered when *P* < .05.

## Results

A total of 51 diabetic patients and 25 healthy control participated in this ABI study. There were 26 patients in group A (with calcified leg arteries), 25 patients in group B (with non-calcified leg arteries), and 25 in group C (non-diabetic with non-calcified leg arteries) healthy volunteers. Table 1, shows the subjects characteristics. The mean age (±SD) for all participants was 64.30 ± 7.1 years (range, 50–82). There was no significant difference in gender, age or BMI between all groups (*P* > .05).

**Table 1:** Subject’s characteristics for all groups

Parameter/Group	Group A	Group B	Group C
	Diabetic patients with calcified limb arteries	Diabetic patients with non-calcified limb arteries	Healthy volunteers
Number Patients/Legs	26/52	25/50	25/50
Sex Female/Male	9/17	9/16	10/15
Age (Mean ± SD, y)	66.8 ± 8.6	63.2 ± 6.8	62.7 ± 4.5
BMI (Mean ± SD,kg/m2)	29 ± 5	28.5 ± 4.5	27 ± 4
Fasting Plasma Glucose (Mean ± SD, mmol/l)	8.49 ± 1.13	7.03 ± 0.52	5.41 ± 0.40
HbA1c (Mean ± SD, %)	7.72 ± 0.62	6.62 ± 0.44	5.10 ± 0.30
Pedal pulse			
Strong	10	50	50
Weak	42	0	0
Not felt	0	0	0
Risk Factors for PAD			
Smoker	10	9	0
Dyslipidemia	22	18	0
Hypertension	26	23	0
Renal Impairment/Insufficiency	3	0	0
Medication History			
Insulin injections	19	7	0
Hypoglycemic drugs	7	18	0
Antilipid drugs	22	18	0
Antihypertensive drugs	26	24	0

Before starting the study, reproducibility and validity of the ABI measurements by the oscillometric device (boso ABI-system 100) was tested in 8 patients (16 limbs). The intraclass correlation coefficients (ICC) were >0.85 indicating very good reliability of the oscillometric device for ABI measurements for all the measured parameters.

Thereafter, the three study groups were examined by the same device. Details of mean ± SD (range) systolic and diastolic blood pressures in the upper and lower limbs as measured by the device in all groups are summarized in Table 2.

**Table 2:** Mean ± SD (range) for systolic/diastolic arm and leg blood pressures (s = systolic; d-diastolic, mmHg) for all groups and for both leg sides

Parameter/Group	Group A	Group B	Group C
	Diabetic patients with calcified limb arteries	Diabetic patients with non-calcified limb arteries	Healthy volunteers
Arm Pressures			
Right arm-s	154.6 ± 20.4 (120-210)	144.1 ± 14.5 (120-163)	132.3 ± 6.14 (119-143)
Right arm-d	92.8 ± 8.3 (80-110)	89.1 ± 7.8 (72-102)	79.4 ± 3.9 (72-90)
Left arm-s	152.8 ± 20.6 (126-215)	142.3 ± 15.1 (120-167)	130.7 ± 5.6 (120-140)
Left arm-d	92.1 ± 8 (80-110)	89.3 ± 6.5 (76-102)	78.9 ± 3.7 (70-89)
Leg Pressures			
Right Leg-s	201.4 ± 26 (154-260)	156.6 ± 14.5 (134-175)	142.4 ± 6.8 (130-153)
Right Leg-d	101.1 ± 8.5 (86-117)	96.1 ± 6.5 (85-107)	84 ± 4.4 (78-100)
Left Leg-s	201 ± 25 (149-270)	156.2 ± 14 (134-178)	142.1 ± 6.2 (131-150)
Left Leg-d	100.2 ± 8.3 (88-115)	96.1 ± 6.6 (86-107)	84.6 ± 3.9 (77-97)

The ABI-s and ABI-d measurements are summarized in Table 3. In group A, ABI-s mean ± SD (range) was 1.3 ± 0.10 (1.18-1.58) and 1.28 ± 0.09 (1.14-1.53) on the right and left legs respectively. In group B, the ABI-s mean ± SD (range) was 1.07 ± 0.05 (1-1.16) and 1.07 ± 0.05 (1.01-1.16) on the right and left legs respectively. In group C, the ABI-s mean ± SD (range) was 1.06 ± 0.05 (1-1.16) and 1.06 ± 0.04 (1.01-1.15) on the right and left legs respectively.

**Table 3:** Summary of mean ± SD for ankle-brachial pressure index systolic/diastolic (ABIs and ABI-d) and the delta (ABI-s minus ABI-d) for all groups and for both leg sides (Repeated measures ANOVA test; Group A vs group B and group A vs group C for each leg (*P* < .001); Group B vs group C (*P* = .543 for right leg & *P* = .720 for left leg)

Parameter/Group	Group A		Group B		Group C	
	Diabetic patients with calcified limb arteries		Diabetic patients with non-calcified limb arteries		Healthy volunteers	
ABI-s	1.30 ± 0.10	1.28 ± 0.09	1.07 ± 0.05	1.07 ± 0.05	1.06 ± 0.05	1.06 ± 0.04
ABI-d	1.07 ± 0.05	1.06 ± 0.04	1.06 ± 0.05	1.06 ± 0.04	1.05 ± 0.04	1.05 ± 0.04
Delta (ABI-s minus ABI-d)	0.22 ± 0.09	0.22 ± 0.08	0.01 ± 0.02	0.01 ± 0.02	0.02 ± 0.02	0.01 ± 0.02

ABI-d in group A mean ± SD (range) was 1.07 ± 0.05 (1-1.17) and 1.06 ± 0.04 (1.10-1.17) on the right and left legs respectively. In group B, ABI-d mean ± SD (range) was 1.06 ± 0.05 (1-1.14) and 1.06 ± 0.04 (1-1.13) on the right and left legs respectively. In group C, ABI-d mean ± SD (range) was 1.05 ± 0.04 (1.01-1.14) and 1.05 ± 0.04 (1.01-1.13) on the right and left legs respectively.

A generalized linear model carried out in which the independent variable is group (A/B/C) showed statistically significantly different between delta for ABI-s & ABI-d for right and left leg (*P* < .001) with R-squared = 0.761 & 0.802 for right and left leg, respectively. In addition, the Post-Hoc test shows that the difference is between group A and group B (*P* < .001), group A and group C (*P* < .001), whereas in group B and group C these indexes were not statistically significant for right leg (*P* = .854) and for left leg (*P* = .844). In addition, repeated measures ANOVA test showed that the group factor is affecting ABI’s measures with (*P* < .001) between groups and within groups for ABI’s (s and d) for each leg side. The pairwise comparisons was statistically significant between group A versus groups B & C for all the parameters (*P* < .001) but it was not significant between group B & C (*P* = .543 for right leg& *P* = .720 for left leg).

**Fig. 1:**
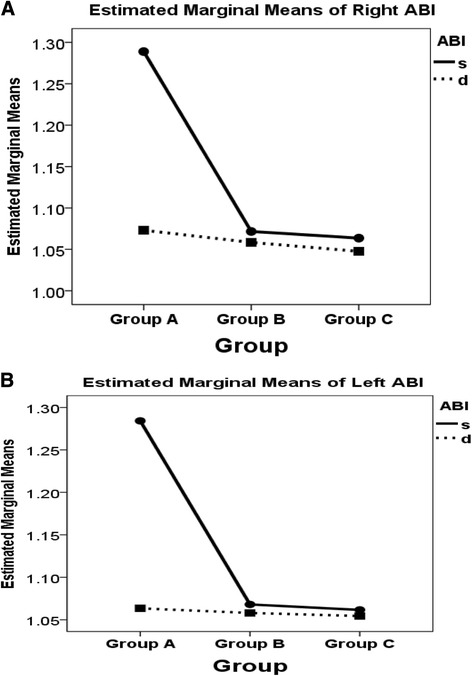
**a-b.** Estimated marginal means for (**a**) right leg & (**b**) for left leg for ABI-s & ABI-d for all groups

It seems that the ABI-d is different from ABI-s value in group A patients with calcified vessel walls and there is no much difference in group B patients and group C volunteers with non-calcified vessel walls. It is noted also that when the arteries were calcified (group A), the difference between ABI-s and ABI-d was higher than the difference between ABI-s and ABI-d when the arteries were not calcified (group B and group C). The results are shown in Table 3 & Fig. 1a-b.

## Discussion

The relationship between ABI and diabetes has drawn the attention of many scientists. To our knowledge, there is no article in literature about measuring the diastolic pressure to calculate the ABI in patients with diabetes with calcified leg arteries instead of measuring the systolic pressure. In this study, we aimed at evaluating the feasibility of using the diastolic (ABI-d) instead of the systolic (ABI-s) in diabetic patients with calcified incompressible leg arteries. We used the automated oscillometric technique to measure systolic and diastolic ABI in diabetic patients who had calcification and those who did not have calcification in their lower limb arteries.

A cutoff of ≤ 0.90 was shown to have a high accuracy rate and has proven to be a good indicator of the presence of PAD even in asymptomatic patients [10, 17]. In a meta-analysis, this index was shown to be a good predictor of cardiovascular disease and mortality [8]. Unfortunately, ABI-s loses its accuracy in the presence of calcified uncompressible arteries in patients with diabetes mellitus, chronic renal disease, and in old patients. In this group, the ABI-s will be falsely elevated precluding its benefit in diagnosis or follow-up of patients with PAD. To overcome this problem, some investigators tried measuring the Toe-Brachial Index (TBI) in patients with uncompressible arteries on the assumption that the severity of arterial calcification is less in the digital arteries. Brooks B. et al measured the ABI and TBI in 174 diabetics and 53 controls, they concluded that in the majority of patients with diabetes, assessment of TBI conveys no advantage over ABI [18]. This is further confirmed by Sahli D. et al who found in an unmatched groups (healthy controls, type 1, and type2 diabetics) that overall tibial pressure, TBI and ABI were similar between all groups [19]. Furthermore, Williams D. et al [20] found that the TBI had lower sensitivity and specificity than the traditional ABI and a recent 2013 publication by Hoyer et al concluded their study on the use of TBI in the diagnosis of PAD by stating that “Although several guidelines and reviews of PAD diagnostics recommend a TBI <0.70 as a cutoff. [21] In a very recent publication (Quong et al, 2016), it has been stated that TBI cutoff of 0.60–0.70 is not well referenced. These authors concluded that “further studies are recommended to determine if the threshold for diagnosis of peripheral arterial disease based on TBI should be raised” [22]. However, other authors concluded studies that TBI is proved to be a sensitive test but ABI is a specific test for PAD [23, 24]. Toe pressure measurement is technically demanding, it requires temperature control of the body, toe and foot with special foot positioning; which may affect precise pressure measurements [21].

Based on the above, it is clear that TBI measurement is still not fully established as a reliable test for PAD and therefore, we cannot say today that we do not need a new parameter to improve or noninvasive assessment of PAD in the setting of calcified arteries. Therefore, we opted in this study to investigate as to whether the ABI-d would be of value in patients with calcified uncompressible lower limb arteries. We have chosen to use the oscillometric technique to measure the ABI, because with this equipment both the ankle diastolic and systolic pressures are simultaneously recoded. It is very simple as it only requires wrapping of the cuffs around the ankle and arm then with a push of a button the machine starts recording. It is operator-independent and can measure the ankle systolic and diastolic pressures even in the presence of total or partial arterial incompressibility [14–16]. In a previous study Sella-Cunha SX et al showed that by using this technique, measurement of the diastolic ABI (ABI-d) increased the sensitivity of the systolic ABI (ABI-s) from 52 to 77 % [16]. In the present study we have shown that the boso ABI machine measurement is repeatable, however oscillometric machines have been previously shown to be inaccurate in nearly 20 % of the time [17].

In this study we compared two well-matched groups of diabetic patients and healthy volunteers, those with calcified arteries with those who did not have arterial calcification. The results showed that in patients with calcified arteries the ABI-d was significantly lower than the ABI-s (*P* < .001) and there was no significant difference between these two indices in diabetics who did not have calcified arteries (*P* > .05). This significant difference between ABI-s and ABI-d in patients with calcified leg arteries (group A) might be due to uncompressible leg arteries. Accordingly, we would suggest that in patients with falsely high ABI-s (>1.3-1.5), to consider measuring the diastolic ankle brachial index instead of the systolic as a complementary parameter to get a better objective view of the arterial blood flow in the lower limbs. These results confirm those of Salles-Cunha et al who found that when screening for PAD, ABI-d improved the sensitivity of detection especially in patients with calcified tibial arteries [16]. Therefore, we propose that the ABI-d could be a valid measurement for those with calcified arteries, we are proposing that the same ABI-s cutoff values of .90 or greater could apply.

Limitations of this study is that we don’t have patients with supra-normal ABI or non-compressible vessels. As these patients would be the patient population that may benefit from a more accurate measurement. The other limitation of this study is, to prove ABI-d is comparable or reliable measure to ABI-s we could compare the automated boso ABI machine measurement to a mercury sphygmomanometer and continuous Doppler device [25]. This would have prolonged the time of the examination and the major aim of the study was to obtain the ABI-s and ABI-d values for comparison between all the groups and further studies are need to explore this issue. In addition, the relatively small sample size and the fact that the patients were chosen on the basis that none has had any signs or symptoms of flow-limiting arterial disease, this decision was to reduce, as far as possible, any confounders. Larger studies which may include patients with symptoms of PAD would further elucidate the value of measuring the ABI-d instead of the ABI-s.

## Conclusions

In conclusion, this study preliminary suggests at some point that the diastolic pressure index may be helpful, and therefore, ABI-d can be used as a complementary measure instead of ABI-s in falsely elevated ABI caused by partial or complete incompressibility in diabetic feet patients with calcified lower limb arteries for assessment of peripheral arterial disease. Further studies are needed to explore how the technique actually does in predicting calcified arteries. Also a study that tested for validity of the technique would be more interesting to the researchers in the field of vascular disorders.
